# A Genetics Pearl for Counseling Patients with Epsilon-Sarcoglycan Myoclonus-Dystonia

**DOI:** 10.5334/tohm.783

**Published:** 2023-08-22

**Authors:** Alissa S. Higinbotham, Suzanne D. DeBrosse, Camilla W. Kilbane

**Affiliations:** 1 Neurological Institute, University Hospitals Cleveland Medical Center, US; 2 Case Western Reserve University, US; 3 Center for Human Genetics, University Hospitals Cleveland Medical Center, US

**Keywords:** Myoclonus, dystonia, myoclonus-dystonia, movement disorders, genetic counseling, education

## Abstract

**Background:**

Epsilon-sarcoglycan (SGCE) myoclonus-dystonia is autosomal dominant (AD) with reduced penetrance due to maternal imprinting 95% of the time. Patients may lack family history delaying diagnosis and treatment. Additionally, counseling patients on their risk of passing on the variant differs for females versus males.

**Case Report:**

A woman in her thirties with typical phenotype of myoclonus-dystonia but lacking an AD pedigree was found to have a pathogenic variant in the SGCE gene. She was counseled that her daughters each have a 2.5% chance of expressing the phenotype.

**Discussion:**

Understanding the genetics of SGCE-myoclonus-dystonia enables effective genetic counseling and arrival at a timely diagnosis and treatment.

**Summary:**

In an era of advancing genetic analysis and precision medicine-based treatments, neurologists will be faced with increasing responsibility to properly counsel patients on the results of genetic testing. This case highlights a genetics pearl for counseling patients with epsilon-sarcoglycan myoclonus-dystonia, an autosomal dominant condition with penetrance differing by sex.

## Introduction

Advances in genetic testing and analysis have provided clinicians and patients with diagnostic clarity, eligibility for clinical trials and, in some cases, a precision medicine-based treatment approach [1]. However, with increasing genetic information comes increasing responsibility to properly counsel patients on the implications of their genetic test result for themselves and for their family members. Clinicians do not always have the luxury of a genetic counselor, especially when faced with impromptu questions in the clinic from patients and family members, and thus basic knowledge of the genetics of the suspected disorder is important. The following case provides a ‘genetics pearl,’ or important genetic counseling tip, for patients with epsilon-sarcoglycan (SGCE) myoclonus-dystonia.

Myoclonus-dystonia is a syndrome characterized by isolated myoclonus or myoclonus with dystonia that predominantly affects the upper body. The dystonic phenomenology generally presents as cervical dystonia or writer’s cramp, but lower limb dystonia can occur early in the disease course and then subsequently abate or even remit later in life [2]. The myoclonus also predominantly affects the upper body and is exquisitely alcohol-responsive which can lead to alcohol dependence [2]. Additional non-motor symptoms, present in half of patients with myoclonus-dystonia syndrome (MDS), include depression, anxiety disorders, and obsessive-compulsive disorder [3]. Epilepsy in MDS has also been reported [4]. It is debated whether the psychiatric symptoms are a consequence of the disease process of SGCE myoclonus-dystonia or sequela of the motor symptoms, especially since dystonia is increasingly recognized to be associated with non-motor psychiatric symptoms [3,5]. Patients are otherwise neurologically and developmentally normal with a normal magnetic resonance image (MRI) of the brain as per the diagnostic criteria for definite myoclonus-dystonia [6].

The diagnostic criteria also include a positive family history since myoclonus-dystonia is classically an autosomal dominant disorder with up to 50% of patients demonstrating pathogenic variants in the autosomal dominantly inherited SGCE gene. However, the SGCE maternal allele undergoes imprinting (methylation and silencing) 95% of the time resulting in reduced penetrance [6]. Non-imprinted pathogenic variants are expected to result in symptoms in all cases, but the severity of such symptoms can vary, possibly due to epigenetic and/or environmental factors [7]. Reduced penetrance due to maternal imprinting can mask an autosomal dominant inheritance pattern as demonstrated in the following patient case.

## Case description

**Figure 1:**
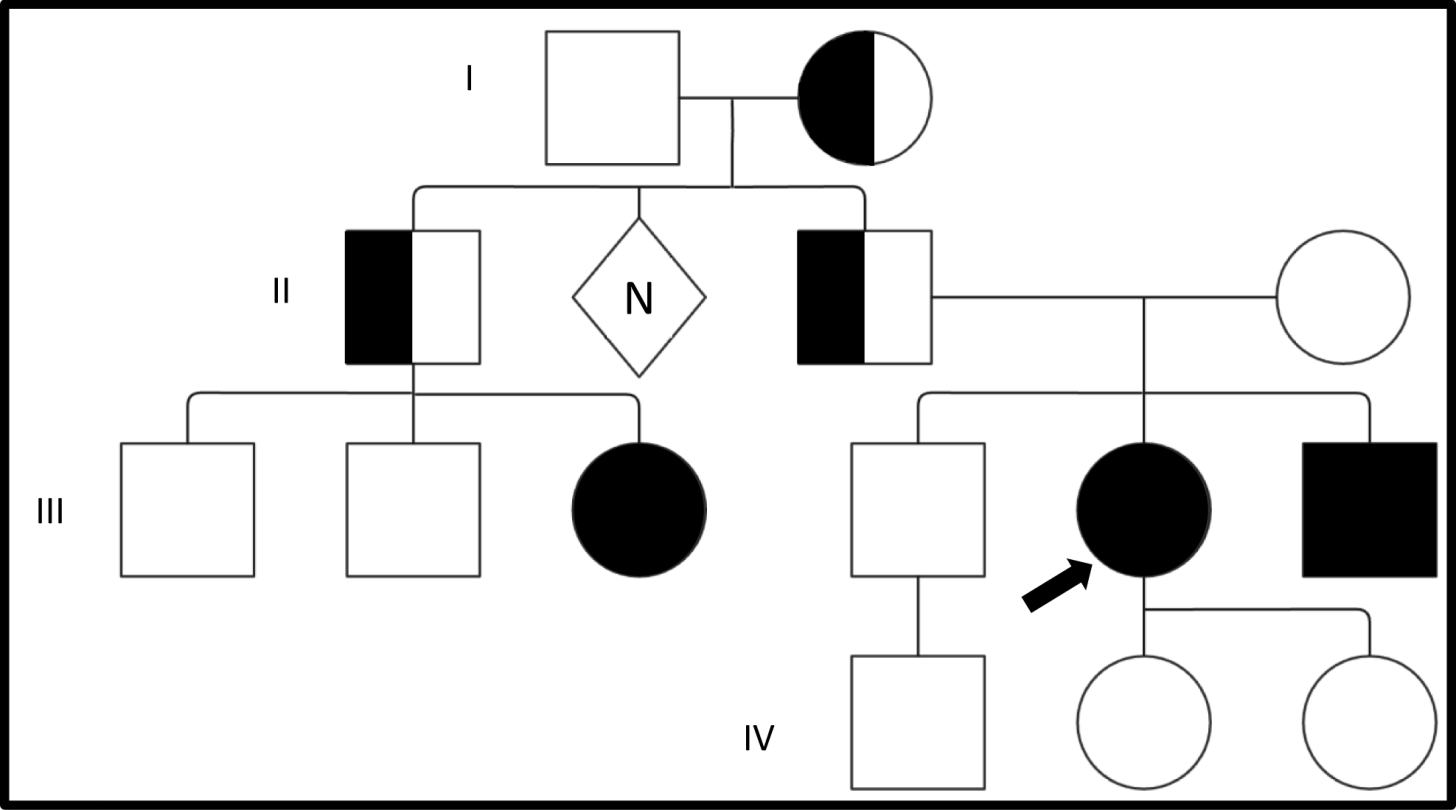
**SGCE myoclonus-dystonia: autosomal dominant transmission with reduced penetrance.** The patient pedigree was simplified for privacy. The patient is indicated by the arrow. The patient’s brother and female cousin were symptomatically affected with myoclonus but did not undergo confirmatory genetic testing. The patient’s grandmother (generation I) was likely the carrier of a maternally imprinted pathogenic SGCE allele that upon transmission to generation II was again maternally imprinted and silenced. However, once paternally transmitted by the patient’s father and uncle, the autosomal dominant transmission pattern re-emerges in generation III. In generation IV, there is only a 2.5% chance each of the patient’s young daughters will be affected in their lifetime due to maternal imprinting.

The patient is a woman in her thirties who developed jerky arm movements at 3 years of age which progressed over time, worsened with action, and improved with alcohol. Additionally, she developed a jerky tremor of the neck as well as difficulty writing. At 8 years of age, she experienced generalized tonic-clonic seizures that resolved after initiation of levetiracetam. Subsequent brain MRI and electroencephalograms (EEGs) were unremarkable. However, she later developed paroxysmal non-epileptic spells (PNES) confirmed in an epilepsy monitoring unit admission. Her family history was remarkable for similar jerky upper body movements in her brother and a female cousin, but in no family members in prior generations (Figure 1). Her neurological examination revealed left laterocollis, left torticollis with dystonic head tremor, mild right dystonic finger flexion, a dystonic grip on the pen when writing, and proximal upper extremity myoclonus that worsened with actions like writing, but was not stimulus sensitive. The remainder of her neurological exam was unremarkable.

Despite lack of autosomal dominant inheritance pattern in her pedigree, clinical suspicion was high for myoclonus-dystonia, so she underwent single gene testing which was positive for a pathogenic variant (Invitae, NM_003919.2: c.835_839del (p.Thr279Alafs*17)) in the epsilon-sarcoglycan (SGCE) gene. Levetiracetam prescribed for epilepsy was ineffective for her myoclonus, so she was started on zonisamide and underwent botulinum toxin injections for cervical dystonia. Over the last 3 years at subsequent follow-up visits, she has reported mild symptomatic improvement with treatment interventions.

**Figure 2:**
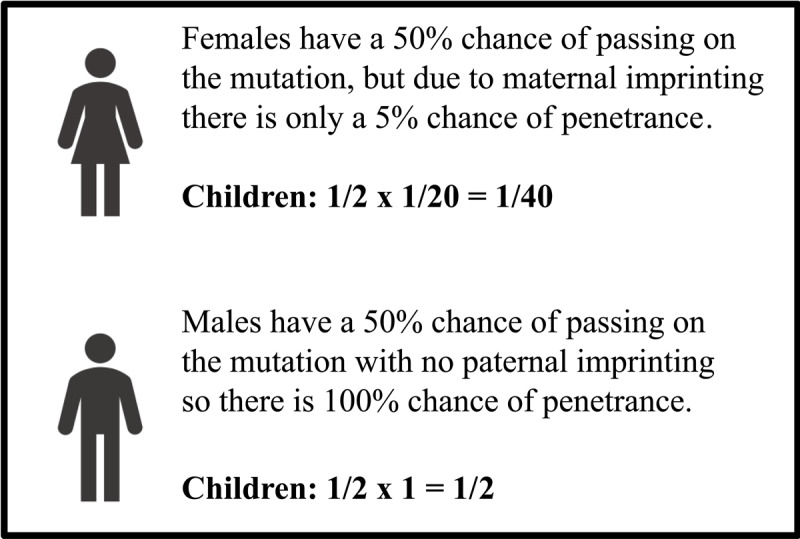
**A genetics pearl for counseling patients with SGCE myoclonus-dystonia:** Penetrance of the SGCE pathogenic variant differs for children of male versus female carriers.

Following diagnosis, she inquired about the risk of her asymptomatic preschool-aged and teenage daughters. She was counseled that patients under the age of 18 generally should not undergo predictive genetic testing when it will not change medical management, but that typically myoclonus-dystonia presents in childhood with most patients developing symptoms before the age of 20 [6]. There was a 1 in 2 chance that she passed the pathogenic variant to each daughter, but due to imprinting (silencing) of her maternal allele 95% of the time, there is only a 5% (1 in 20) chance that the pathogenic allele will be expressed if it was inherited. Thus, each daughter’s individual risk of expressing the phenotype is 1 in 2 multiplied by 1 in 20, or 1 in 40 (2.5% chance). If the patient has a son, he will have the same risk of inheriting and expressing the phenotype as her daughters. Since her daughters will also imprint their SGCE allele when they have children, provided they inherited the pathogenic variant, the risk of passing on and expressing the phenotype for each of the patient’s grandchildren is also 1 in 40 (2.5%). However, if the patient has a grandson with the pathogenic variant, his risk of passing on the variant to each of his children, her great-grandchildren, would be 1 in 2 (50%), and those who inherit the variant would be expected to be symptomatic because there is no paternal imprinting, and the paternal copy of the gene would be expressed. Figure 2 depicts and summarizes the counseling provided above as a concise genetics pearl for reference in the clinic. Written informed consent was obtained from the patient for the publication of this manuscript.

## Discussion

This case illustrates a classic clinical presentation of SGCE myoclonus-dystonia, the genetics of SGCE myoclonus-dystonia; autosomal dominant with reduced penetrance when maternally inherited and a genetics pearl on how to counsel patients on the risk of transmission which differs for females versus males as illustrated in Figure 2.

In this case, despite a potentially misleading pedigree, knowledge of the classic myoclonus-dystonia phenotype and the phenomenon of reduced penetrance with maternal transmission enabled a targeted approach to diagnosis with single gene testing, avoiding excessive cost to the patient, incidental positive results, as well as variants of uncertain significance that can complicate diagnosis and genetic counseling and delay effective treatment [8].

Arriving at a timely genetic diagnosis for myoclonus-dystonia syndrome can be important for precision medicine-based treatment, namely deep brain stimulation (DBS) surgery if oral medications and botulinum toxin are ineffective. Both myoclonus and dystonia can respond to DBS in SGCE myoclonus-dystonia with acceptable targets including globus pallidus interna (GPi) and/or ventral intermediate nucleus (VIM) of the thalamus [9,10,11,12]. While patients lacking SGCE pathogenic variants have also been reported to respond to DBS [13], there are some genetic loci associated with myoclonus-dystonia syndrome for which response to DBS is not yet known such as myoclonus-dystonia 15 (DYT15), mapped to a locus on chromosome 18p11with unknown gene [14]. Additionally, there are some genes associated with myoclonus-dystonia syndrome for which an alternative treatment can provide robust response such as a case of dopa-responsive dystonia presenting as myoclonus-dystonia that responded to levodopa [15].

Awareness of effective treatment options is important for SGCE myoclonus-dystonia, because when treatment is delayed, patients may self-medicate with alcohol leading to dependence or develop worsening psychiatric symptoms and quality of life. Additionally, questions of treatment naturally arise following genetic counseling and diagnosis discussions with the patient.

Returning to the patient case, she went through life undiagnosed for 30 years and eventually developed non-motor symptoms of anxiety and paroxysmal non-epileptic spells. These spells have resolved since receiving a genetic diagnosis as well as counseling for what to expect for her children and grandchildren. Her quality of life with current treatments is stable but will continue to be monitored, and DBS could be considered in the future if necessary.

## References

[1] Papadopoulou E, Pepe G, Konitsiotis S, et al. The evolution of comprehensive genetic analysis in neurology: implications for precision medicine. Journal of the Neurological Sciences. 2023; 447(120609). DOI: 10.1016/j.jns.2023.120609 36905813

[2] Roze E, Lang AE, Vidailhet M. Myoclonus dystonia: classification, phenomenology, pathogenesis and treatment. Current Opinion in Neurology. 2018; 31(4): e484–490. DOI: 10.1097/WCO.0000000000000577 29952836

[3] Kim JY, Lee WW, Shin CW, et al. Psychiatric symptoms in myoclonus-dystonia syndrome are just concomitant features regardless of the SGCE gene mutation. Parkinsonism and Related Disorders. 2017; 42: e73–77. DOI: 10.1016/j.parkreldis.2017.06.014 28690014

[4] O’Riordan S, Ozelius LJ, de Carvalho Aguiar P, et al. Inherited myoclonus-dystonia and epilepsy: further evidence of an association? Movement Disorders. 2004; 19(12): e1456–1459. DOI: 10.1002/mds.20224 15389977

[5] Peall KJ, Smith DJ, Kurian MA, et al. SGCE mutations cause psychiatric disorders: clinical and genetic characterization. Brain. 2013; 136(1): e294–303. DOI: 10.1093/brain/aws308 PMC4052887 23365103

[6] Kinugawa K, Vidailhet M, Clot F, et al. Myoclonus-dystonia: an update. Movement Disorders. 2009; 24(4): e479–489. DOI: 10.1002/mds.22425 19117361

[7] Asmus F, Hjermind LE, Dupont E, et al. Genomic deletion size at the epsilon-sarcoglycan locus determines the clinical phenotype. Brain. 2007; 130(10): e2736–2745. DOI: 10.1093/brain/awm209 17898012

[8] Salunkhe M, Agarwal A, Faruq M, et al. Genetic testing in neurology: what every neurologist should know. Annals of Indian Academy of Neurology. 2022; 25(3): e350–353. DOI: 10.4103/aian.aian_855_21 PMC9350807 35936600

[9] Kosutzka Z, Tisch S, Bonnet C, et al. Long-term GPi-DBS improves motor features in myoclonus dystonia and enhances social adjustment. Movement Disorders. 2019; 34(1): e87–94. DOI: 10.1002/mds.27474 30302819

[10] Krause P, Koch K, Gruber D, et al. Long-term effects of pallidal and thalamic deep brain stimulation in myoclonus dystonia. European Journal of Neurology. 2021; 28(5): e1566–73. DOI: 10.1111/ene.14737 33452690

[11] Zhang YQ, Wang JW, Wang YP, et al. Thalamus stimulation for myoclonus dystonia syndrome: five cases and long-term follow-up. World Neurosurgery. 2019; 122: e933–39. DOI: 10.1016/j.wneu.2018.10.177 30419400

[12] Wang X, Yu X. Deep brain stimulation for myoclonus dystonia syndrome: a meta-analysis with individual patient data. Neurosurgical Review. 2021; 44: e451–62. DOI: 10.1007/s10143-019-01233-x 31900736

[13] Sidiropoulos C, Mestre T, Hutchinson W, et al. Bilateral pallidal stimulation for sarcoglycan epsilon negative myoclonus. Parkinsonism and Related Disorders. 2014; 20(8): e915–18. DOI: 10.1016/j.parkreldis.2014.04.017 24812007

[14] Alterman RL, Filippidis AS. Genetic subtypes and deep brain stimulation in dystonia. Movement Disorders Clinical Practice. 2018; 5(4): e351–453. DOI: 10.1002/mdc3.12660 PMC6336377 30838292

[15] Sharma P, Holla VV, Gurram S, et al. Myoclonus-dystonic presentation of childhood onset DYT-GCH1: a report from India. Journal of Movement Disorders. 2023; 16(1): e101–103. DOI: 10.14802/jmd.22106 PMC9978263 36628429

